# Timing for Intravenous Injection of Mesenchymal Stem Cells to Enhance Fracture Healing

**DOI:** 10.3390/jfb17080361

**Published:** 2026-07-27

**Authors:** Kang-Il Kim, Gi-Young Jang, Kye-Youl Cho, Myung-Seo Kim, Hyun-Ju Chung, Hyun-Mi Cho, Ki-Hyeok Ku

**Affiliations:** 1 Department of Orthopedic Surgery, Kyung Hee University Hospital at Gangdong, Kyung Hee University School of Medicine, Seoul 05278, Republic of Korea; khuknee@gmail.com (K.-I.K.); remomg725@naver.com (G.-Y.J.); 84g-t@hanmail.net (M.-S.K.); 2 Center for Advanced Regenerative Medicine, Kyung Hee University Hospital at Gangdong, Seoul 05278, Republic of Korea; hjchung@khnmc.or.kr (H.-J.C.); dream-hahaha@hanmail.net (H.-M.C.); 3 Department of Medicine, Graduate school of Kyung Hee University, Seoul 02453, Republic of Korea; dros@daum.net

**Keywords:** systemic intravenous injection, optimal timing, allogeneic, mesenchymal stem cells, fracture healing

## Abstract

When delivered intravenously, mesenchymal stem cells (MSCs) enhance fracture healing and can be injected at various time points, but the optimal timing remains unclear. This study aimed to clarify the optimal timing of MSC delivery to enhance the healing. For-ty-nine Wistar rats with femoral shaft fractures were randomized according to injection timing: immediately after fracture (group A, *n* = 14), 24 h after fracture (group B, *n* = 14), 7 days after fracture (group C, *n* = 14), and control group (group D, *n* = 7). Allogeneic bone-marrow-derived MSCs (5.0 × 10^6^ cells) were administered intravenously. Rats were euthanized at 6 weeks post-fracture for analysis. In each group, seven rats were evaluated for new bone formation and histological examination. Another seven rats in group A, B, and C were evaluated for VEGF, TGF-β1, and BMP-2 expression. Group C showed significantly higher new bone formation than groups A, B and D (*p* = 0.017, *p* = 0.003, and *p* = 0.005). Histological grades were higher in group C than in groups D (*p* = 0.018). In Western blot analysis, VEGF and BMP-2 were higher in group C (*p* < 0.05). Real-time polymerase chain reaction revealed higher VEGF and TGF-β1 RNA expression in group C (*p* < 0.05). MSCs administration at 7 days post-fracture is suggested to be the optimal timing for intravenous injection to enhance fracture healing in a rat long bone fracture model.

## 1. Introduction

Mesenchymal stem cells (MSCs) are plastic-adherent mesenchymal stromal cells that express specific surface molecules and exhibit multipotent differentiation into osteoblasts, adipocytes, and chondroblasts [1,2]. These cells have the potential to enhance osteogenesis, angiogenesis, and immunomodulation [3,4,5]. MSCs can be harvested from various sources such as adipose tissue, umbilical cord, peripheral blood, and bone marrow [3]. Among these sources, bone-marrow-derived MSCs have high affinity to differentiate into the osteoblastic lineage [3,6].

Several animal studies have reported that MSCs enhance fracture healing [7,8]. In fracture models, MSCs are administered by direct injection into the fracture site, injection into fracture site using a scaffold, or systemic intravenous delivery [3,9]. Wilson et al. injected allogeneic adipose-derived MSCs either directly or intravenously into a bone defect in the swine ramus [8]. They reported increased bone healing in the group injected with MSCs. Kim et al. also injected human MSCs directly into femoral shaft fractures in rats and reported that the optimal concentration of MSCs to enhance fracture healing was at least 5.0 × 10^6^ MSCs [4].

The secondary bone healing process begins with an acute inflammatory response that peaks within the first 24 h and concludes after 7 days, followed sequentially by the reparative and remodeling phases [3,10]. This healing process suggests that the efficacy of MSC-mediated enhancement of fracture healing may depend on the timing of MSC delivery. Systemic intravenous administration enables flexible timing, in contrast to direct local injection. To the best of our knowledge, only two studies have evaluated the timing of MSC injection for fracture healing. Dreger et al. compared systemic intravenous MSC delivery at 24 h versus 3 days after fracture in mice and identified the 24 h time point as optimal [11]. Wang et al. compared tail vein injections on days 1, 7, and 14 after fracture in a murine model. They found that day 7 was the optimal injection timing [12]. However, there is no consensus on when the optimal timing of intravenous MSC delivery to enhance fracture healing in a long bone fracture model of the rat is. Thus, in this study, we aimed to determine the optimal timing of intravenous MSC administration in such a model.

## 2. Materials and Methods

### 2.1. Animal Model

Forty-nine adults male Wistar rats (7 weeks old, 190–250 g) were obtained from Orient Bio Inc. (Seongnam-si, Gyeonggi-do, Republic of Korea). The rats were provided ad libitum access to food and water and were housed in a breeding facility with a controlled environment within our research institute. To minimize stress related to environmental changes, the rats arrived at the institute 1 week before surgery. The rats underwent surgery for a fracture model at 8 weeks of age (220–280 g). As exclusion criteria, animals experiencing unexpected severe pain and inability to eat or move normally because of pain or showing more than 20% loss in body weight were humanely euthanized.

All experimental procedures involving animals adhered to the requirements of the Institutional Animal Care and Use Committee of the Clinical Research Institute. The ethics committee of Kyung Hee University Hospital at Gangdong granted final approval (KHNMC AP 2023-17).

Rats and mice, members of the Muridae family, are widely used in medical research because they are mammals, small, inexpensive, easy to maintain, reproduce quickly, and share a high degree of homology with humans. Orthopedic trauma research commonly uses rats rather than mice because their larger size facilitates the creation and study of surgical fracture models.

### 2.2. Preparation of Allogeneic MSCs

Human MSCs are now commercially produced and widely accessible. Several rat studies have used human MSCs instead of allogeneic rat MSCs because of this convenience. However, human MSC transplantation in rats presents an inherent risk of xenogeneic immune rejection. Allogeneic rat MSCs would be more ideal, yet isolating and expanding murine bone-marrow-derived MSCs remains challenging because of their low content in the bone marrow [13]. Recent reports have described successful isolation and expansion of allogeneic rat MSCs and have proposed culture protocols. These cells have already been used in several studies [12].

In the animal laboratory affiliated with the Department of Orthopedic Surgery in our hospital, our team successfully isolated and cultured allogeneic rat MSCs. All procedures were performed aseptically to ensure sterility [14]. Bone marrow was flushed and collected from 7-week-old male Wistar rats following established protocols [13,14,15]. Briefly, rats were euthanized and sterilized. The femur and tibia were carefully dissected, removing muscles, ligaments, and tendons. Bone marrow was extracted from the bone cavity by flushing with a modified culture medium (Dulbecco’s modified Eagle’s medium 84% + 15% fetal bovine serum + 1% penicillin–streptomycin). All three products are manufactured by Thermo Fisher Scientific Inc. Waltham, MA, USA. The medium containing MSCs was then transferred to a Petri dish and incubated at 37 °C in a 5% CO_2_ environment. Neither a cell filter nor a growth factor was used. Nonadherent hematopoietic cells and debris were removed after 24–48 h by replacing the medium. When cultures reached 70–90% confluence, cells were detached from the dish with 0.25% trypsin and split at a ratio of 1:3 during subculture. Passage 3 cells were used for transplantation.

**Figure A1:**
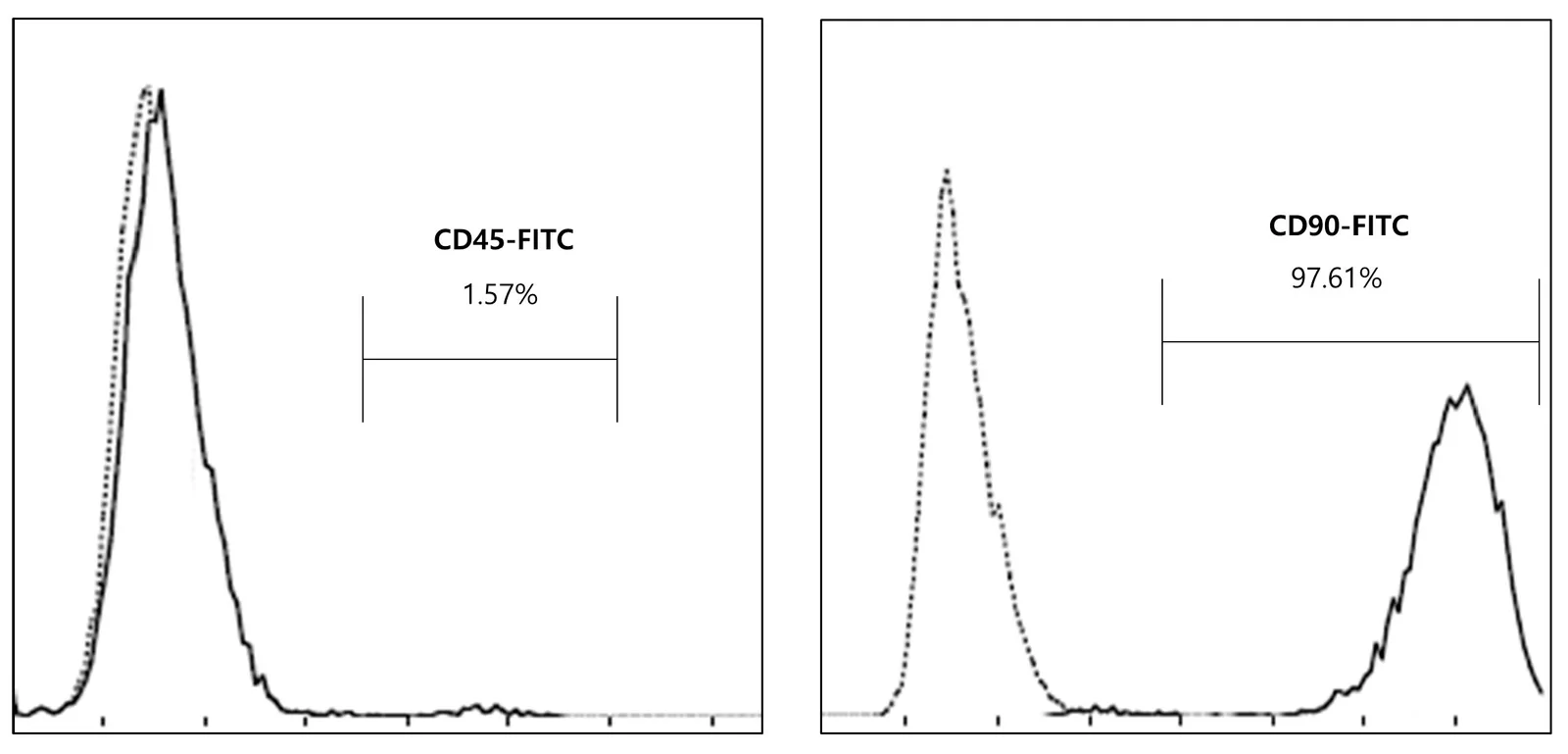
Fluorescence intensity of the MSCs.

**Figure A2:**
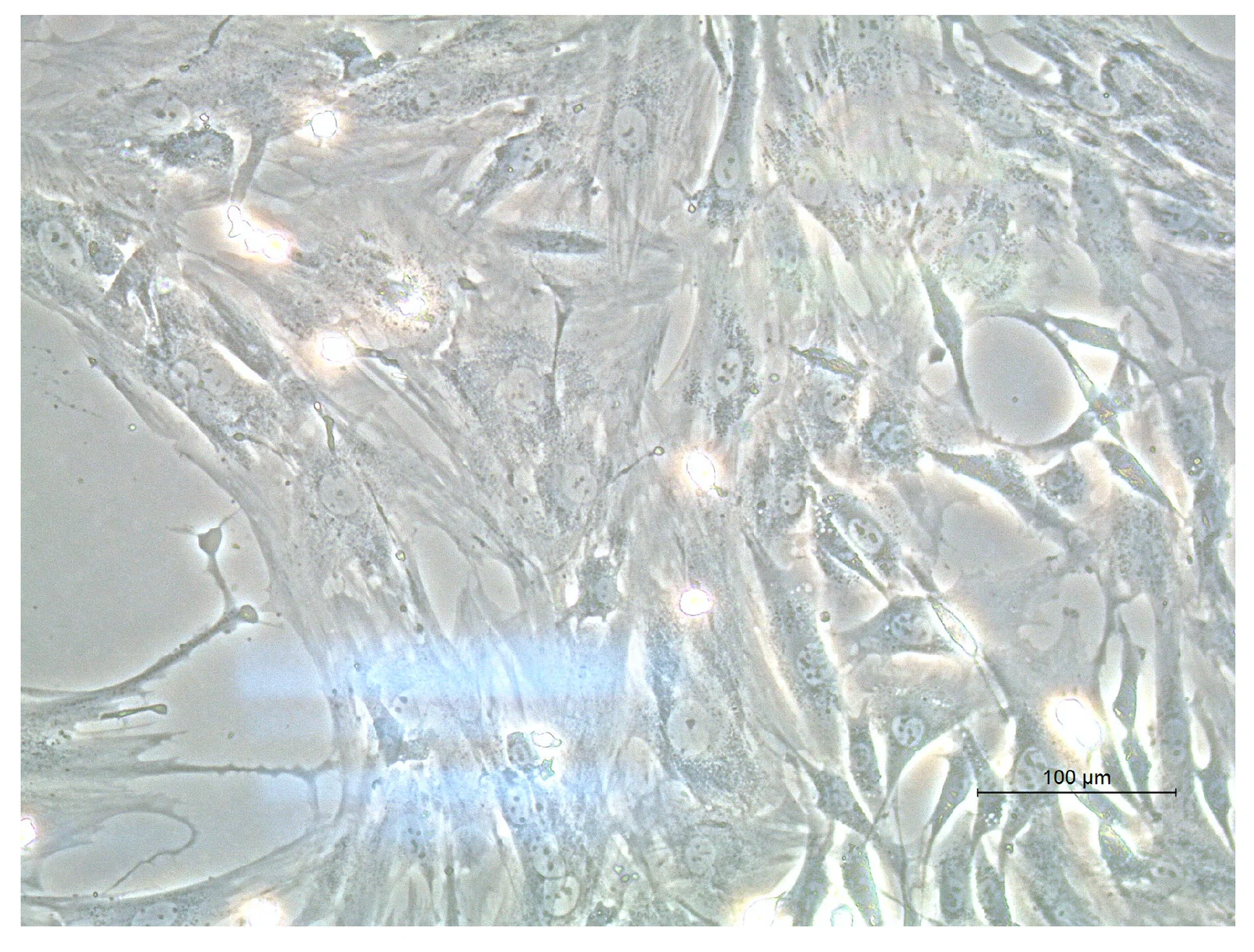
Microscopic photo of the MSCs (20× magnification).

Flow cytometry verified MSC purity using antibodies against specific cluster of differentiation (CD) markers. Among the CD markers defined in 2006 by the International Society for Cellular Therapy to distinguish MSCs, CD90 (catalog no. 11-0900-81; Thermo Fisher Scientific, Waltham, MA, USA) showed positive expression, whereas CD45 (catalog no. 11-0461-82; Thermo Fisher Scientific, Waltham, MA, USA) and CD34 (catalog no. bs-0646R; Bioss Antibodies, Woburn, MA, USA) remained negative [2]. The CD fluorescence intensity and microscopic photo of the MSCs is depicted in Appendix A Figure A1 and Figure A2. The cells also demonstrated differentiation potential into osteocytes, chondrocytes, and adipocytes. To preserve optimal biological viability, MSCs were harvested and counted immediately prior to intravenous injection.

### 2.3. Long Bone Fracture Model and Surgical Technique

Several bones in the murine skeleton, including the femur, tibia, radius, ulna, mandible, and calvarium, have been used to model the fractured environment. Among these, only the femur and tibia provide suitable anatomy for fracture healing studies and accurate biomechanical testing [16,17]. The femur offers several advantages over the tibia, including a straight medullary canal, a larger diameter, the absence of a fibula, and ample soft tissue coverage. The femur also maintains a consistent inner and outer diameter along its length, which enables different diaphyseal fracture sites to produce comparable experimental results [16,17]. Therefore, the femoral shaft was selected for the fracture model in this study. A transverse fracture was created in the right femoral shaft of all rats.

General anesthesia was induced by injecting Zoletil 50 (Virbac, Carros, Alpes-Maritimes, France) 0.1 mL/100 g and Rompun (Bayer, Germany) 0.04 mL/100 g into the abdominal cavity. Deep anesthesia was induced within 3 min. The broad area surrounding the right lower extremity was shaved and sterilized with povidone iodine. A fenestrated drape exposed only the disinfected leg to maintain an aseptic surgical field.

**Figure 1:**
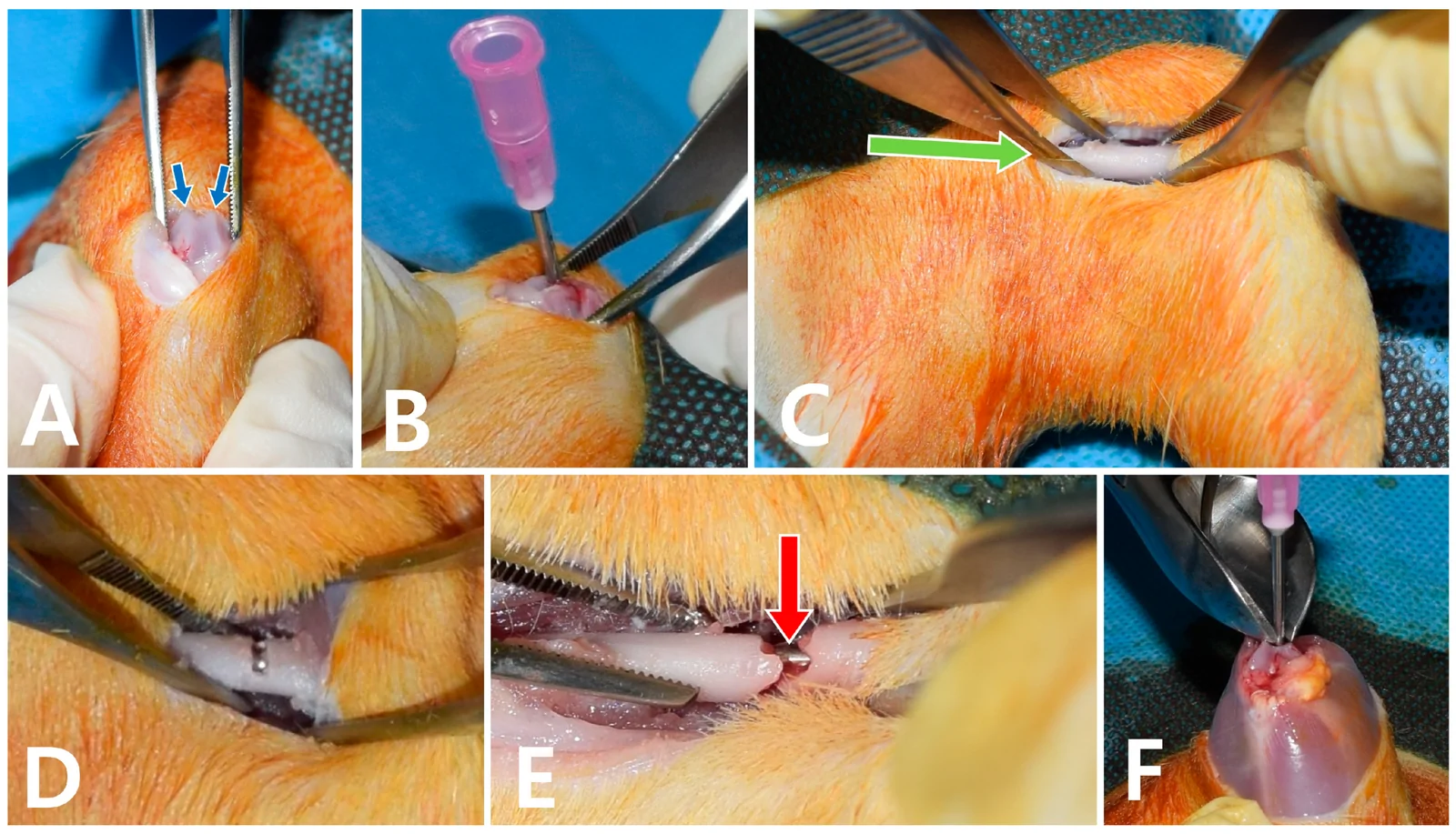
(**A**) Intercondylar groove of the distal femur is exposed. Blue arrows indicate the medial and lateral condyles of the distal femur. (**B**) An 18-gauge needle is retrogradely inserted into the center of the intercondylar groove as an intramedullary nail for fracture fixation. (**C**) A femoral shaft is exposed. Green arrow indicates axis of the femur shaft. (**D**) A cortical discontinuity with a depth of 1 mm is created using the small oscillating thin saw. (**E**) After producing the fracture, the intramedullary nail is re-inserted. Red arrow indicates the 18-gauge needle inserted as the intramedullary nail. (**F**) The end of the needle is cut and advanced into the intramedullary canal.

Preemptive intramedullary nailing was performed before fracturing the bone to facilitate subsequent fixation. An anterior midline incision was made at the knee joint. After dislocating the patella medially or laterally, the knee joint and intercondylar groove of the femur were exposed (Figure 1A). An 18-gauge (1.27 mm diameter) needle was manually rotated in a drilling motion and inserted retrograde into the intramedullary canal through the intercondylar groove (Figure 1B). After advancing approximately 1.8 cm, contact with the proximal femur indicated that the needle was correctly positioned.

On the lateral aspect of the thigh, the femur was palpated, and an incision of approximately 0.7 cm in length was made. Careful dissection minimized periosteal injury, and the femoral shaft was exposed (Figure 1C). A cortical discontinuity was created with the small oscillating thin saw to a depth of 1 mm (Figure 1D). Sterile saline was sprayed onto the fracture site during sawing to reduce heat-related periosteal damage. The cortex had to be cut with caution to avoid contact between the saw and the intramedullary needle. The needle was then retrieved to prepare for fracture creation. A fracture was generated in the femoral shaft using a three-point bending technique [18]. The intramedullary nail was re-inserted into the proximal femur (Figure 1E).

Because the distal end of the needle can protrude into the knee joint and restrict motion, the end was cut and pushed into the intramedullary canal of the femur (Figure 1F). The knee joint capsule and muscular fascia were repaired [16], and the skin was closed. Analgesics and antibiotics were injected intramuscularly into the left side. The rats were allowed unrestricted activity postoperatively.

### 2.4. Study Group Allocation and MSC Intravenous Injection

A total of 49 rats were randomly divided into four groups: immediately after fracture (group A, *n* = 14), 1 day (24 h) after fracture (group B, *n* = 14), 7 days after fracture (group C, *n* = 14), and control group (group D, *n* = 7). Due to limited number of sample throughput that could be processed simultaneously by molecular biology equipment, analyses, except molecular methods, were conducted in the control group. Randomization was performed using computer-generated numbers after completion of the fracture model surgery. MSCs were harvested from culture dishes immediately before intravenous administration. A small aliquot was collected, and cell numbers were determined using Trypan blue staining and a hemocytometer [19]. More than 5 million MSCs were suspended in 0.3 mL phosphate-buffered saline (PBS) and filled in a 1 mL syringe. One syringe per rat was intravenously injected through one of the two tail veins [4]. An independent external researcher performed injections in a blinded manner.

Evaluation required euthanasia of the experimental rats and excision of the fractured femur. The excised femur that underwent micro-computed tomography (micro-CT) scanning was subsequently used for histological grading after sectioning and staining. An excised femur used for micro-CT and histological analysis could not be used for Western blotting or polymerase chain reaction (PCR). Western blotting and PCR are evaluated from scraped bone powder collected from the fracture union site. Bone powder from a single femur sample was sufficient for both Western blot and PCR analyses.

**Figure 2:**
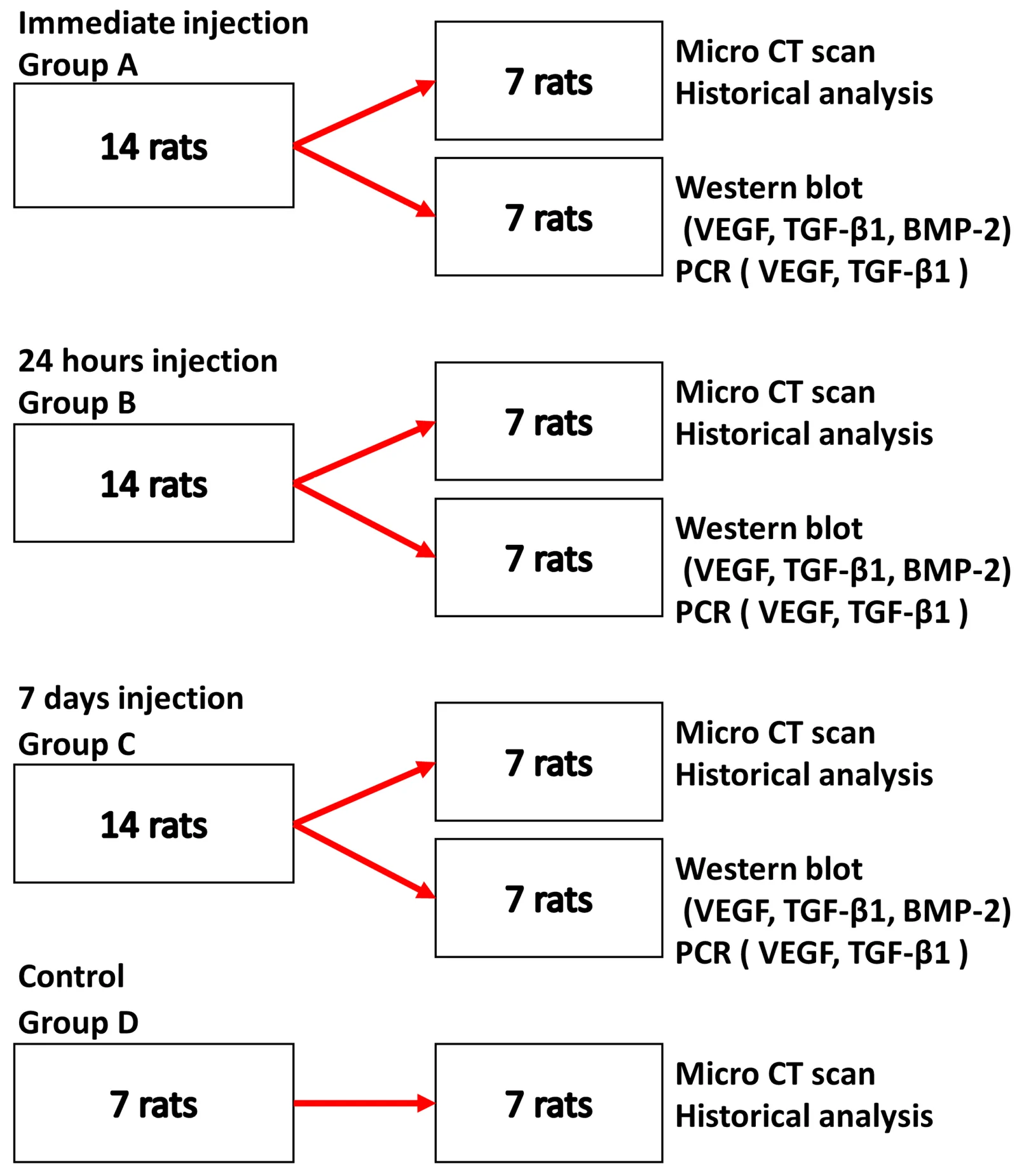
Study design.

At 6 weeks post-fracture, the rats were euthanized for femur specimen harvest. In each group, seven femurs were allocated for micro-CT and histological evaluation. In group A, B, and C, seven femurs were allocated for Western blot and PCR analysis (Figure 2).

### 2.5. Assessment of Fracture Healing

#### 2.5.1. Radiologic Evaluation Through Micro-CT

A 6 mm-long section centered on the fracture site was analyzed [5,12,20]. Pre-existing cortical bone and medullary canal volumes were excluded according to the method described by Wang et al. [12]. The volume of newly formed callus [bone volume (BV)] was measured. Considering inter-individual differences in each rat in femur size, the percentage of BV [percentage BV (PBV), calculated as BV/tissue volume] was used instead of simple BV.

The specimen was extracted with the soft tissue still attached to the fracture site to prevent damage to the bony structure. Subsequently, the intramedullary nail was removed, and the femur was placed in formalin before the CT scan.

The femur specimens were scanned using three-dimensional micro-CT (SkyScan 1172™; Skyscan, Kontich, Belgium or vivaCT 80; Scanco Medical, Brüttisellen, Switzerland) at an 11 μm resolution, 770 ms exposure time, 0.4° rotation step, 55~60 kV, and 167 μA with a 0.5 mm aluminum filter. An independent external analysis facility performed the micro-CT evaluation in a blinded manner.

#### 2.5.2. Histological Examination

Extracted femurs were fixed in 10% formalin. Specimens have been completely decalcified in RDO Rapid Decalcifier^®^ (Apex Engineering Products Corporation, Aurora, IL, USA) at room temperature (21–23°) for 3 days. After decalcification, the demineralized femurs underwent paraffin embedding and serial sagittal sectioning at 5-μm intervals, followed by hematoxylin and eosin (H & E) staining, dehydration, and mounting. Light microscopy (Olympus CX41 microscope, Olympus Company, Tokyo, Japan) was used for slide evaluation. Under 40× and 200× magnification, fracture healing was graded according to a known histological fracture healing scale [21,22]. In this fracture healing scale proposed by Huo et al. in 1991, fibrous tissue was staged as scale 1, cartilage tissue as scale 5, an equal mixture of cartilage and immature bone as scale 7, and union by mature bone as scale 10. An independent external researcher performed slide preparation and fracture healing grading in a blinded manner. Despite sample loss during processing, more than four samples per group were included, allowing Kruskal–Wallis analysis to be conducted.

#### 2.5.3. Western Blot Analysis

Western blot analysis was used to assess protein expression levels of vascular endothelial growth factor (VEGF), transforming growth factor-beta 1 (TGF-β1), and bone morphogenetic protein 2 (BMP-2). VEGF is a protein that regulates angiogenesis. TGF-β1 is associated with MSC migration. BMP-2 is a cytokine associated with osteogenesis. VEGF, TGF-β1, and BMP-2 are “functional” markers of osteogenesis and widely used to evaluate fracture enhancement [4,12].

Excised rat femurs were processed following a known Western blot protocol for femur specimens [4]. Callus tissue was collected, and the total protein was extracted. Protein concentration was measured using a protein assay kit (Thermo Fisher Scientific, Waltham, MA, USA). Total proteins were resolved on 10% sodium dodecyl sulfate–polyacrylamide gel electrophoresis gels, and the separated proteins were transferred onto polyvinylidene fluoride membranes (Sigma-Aldrich, Louis, MO, USA). The membranes were blocked in 5% skim milk in Tris-buffered saline (TBS) containing 0.1% Tween-20 (TBST) for 1 h and then incubated overnight at 4 °C with the following primary antibodies: anti-VEGFA (catalog no. sc7269; Santa Cruz Biotechnology, Dallas, TX, USA, dilution ratio 1:1000), anti-TGF β1 (catalog no. ab215715; Abcam, Cambridge, UK, dilution ratio 1:2000), anti-BMP 2 (catalog no. NBP1-19751; Novus Biologicals, Littleton, CO, USA, dilution ratio 1:5000), and β-actin (catalog no. sc-47778; Santa Cruz Biotechnology, dilution ratio 1:5000). After washing in TBST, the blots were incubated for 1 h with goat anti-mouse horseradish peroxidase-conjugated immunoglobulin G (catalog no. ADI-SAB-100-J and ADI-SAB-300-J; Enzo Life Sciences, Farmingdale, NY, USA) at 37 °C. Bands and band intensities were visualized using the Enhanced Peroxidase Detection Western Reagent Kit^®^ (ELPIS-BIOTECH, Daejeon, Republic of Korea) and an enhanced chemiluminescence system. The relative amount of protein was analyzed using ImageJ^®^ software (version 1.54; National Institutes of Health, Bethesda, MD, USA). β-actin was used as the loading control.

#### 2.5.4. Real-Time Polymerase Chain Reaction (RT-qPCR)

RT-qPCR analysis was used to assess mRNA expression levels of VEGF and TGF-β1. Total RNA was extracted from fresh bone tissue of each group using TRIzol™ reagent (Thermo Fisher Scientific, Waltham, MA, USA) according to the manufacturer’s protocol. RNA quantity and quality were determined using a NanoDrop 2000™ spectrophotometer (Thermo Fisher Scientific). Total RNA was reverse-transcribed using the RevertAid™ First Strand cDNA Synthesis Kit (Thermo Fisher Scientific) according to the manufacturer’s in-structions.

Real-time quantitative polymerase chain reaction (RT-qPCR) was carried out. The re-actions were performed using the StepOnePlus Real-Time PCR System (Applied Biosys-tems, Foster City, CA, USA) and SYBR-Green Real-time PCR Master mix (Thermo Fisher Scientific). The thermocycling conditions consisted of an initial pre-denaturation step at 95 °C for 2 min, followed by 40 cycles of denaturation at 95 °C for 15 s, annealing at 58 °C for 15 s, and extension at 72 °C for 30 s. Expression values were normalized against glyceraldehyde 3-phosphate dehydrogenase (GAPDH) using the 2^-∆∆CT^ method [23]. All experiments were performed in triplicate, and outlier values were removed during data processing [24]. Primer sequences of VEGFA and TGF-β1 used for the analysis are listed in Table 1.

### 2.6. Statistical Analysis

To calculate the sample size, we referred to the previous study conducted by Wang et al. [12]. Cohen’s effect size between days 1 and 7 post-fracture was estimated, and the re-quired sample size was calculated using G*Power software (version 3.1.9.6; Franz Faul, Universität Kiel, Germany) with a significance level of 0.0083 and a power of 0.8. The a priori analysis indicated a total of twelve rats (three per group). After allowing for a 20% dropout, the target increased to 16 (four per group). We ultimately enrolled seven rats per group, which exceeded the calculated requirement.

To compare bone union among the three groups based on the timing of MSC injection, parametric and non-parametric statistical approaches were used. For histological grade, the Kruskal–Wallis method was used, followed by Bonferroni-adjusted pairwise Mann–Whitney U tests as post hoc analysis to identify statistically significant between-group differences. For micro-CT, Western blot, and RT-qPCR data, after a Shapiro–Wilk normality test and Levene’s test for homogeneity of variance, we performed one-way ANOVA with Bonferroni-adjusted post hoc comparisons. Statistical significance was set at α = 0.05 for global tests and adjusted pairwise *p*-values = 0.05, with a 95% CI (i.e., adjusted *p* < 0.05; equivalent to per-comparison α ≈ 0.008 for four comparisons, and α ≈ 0.0167 for three comparisons). SPSS (version 28.0; IBM Inc., Chicago, IL, USA) was used for statistical analyses.

## 3. Results

### 3.1. Radiological Evaluation Through Micro-CT

**Figure 3:**
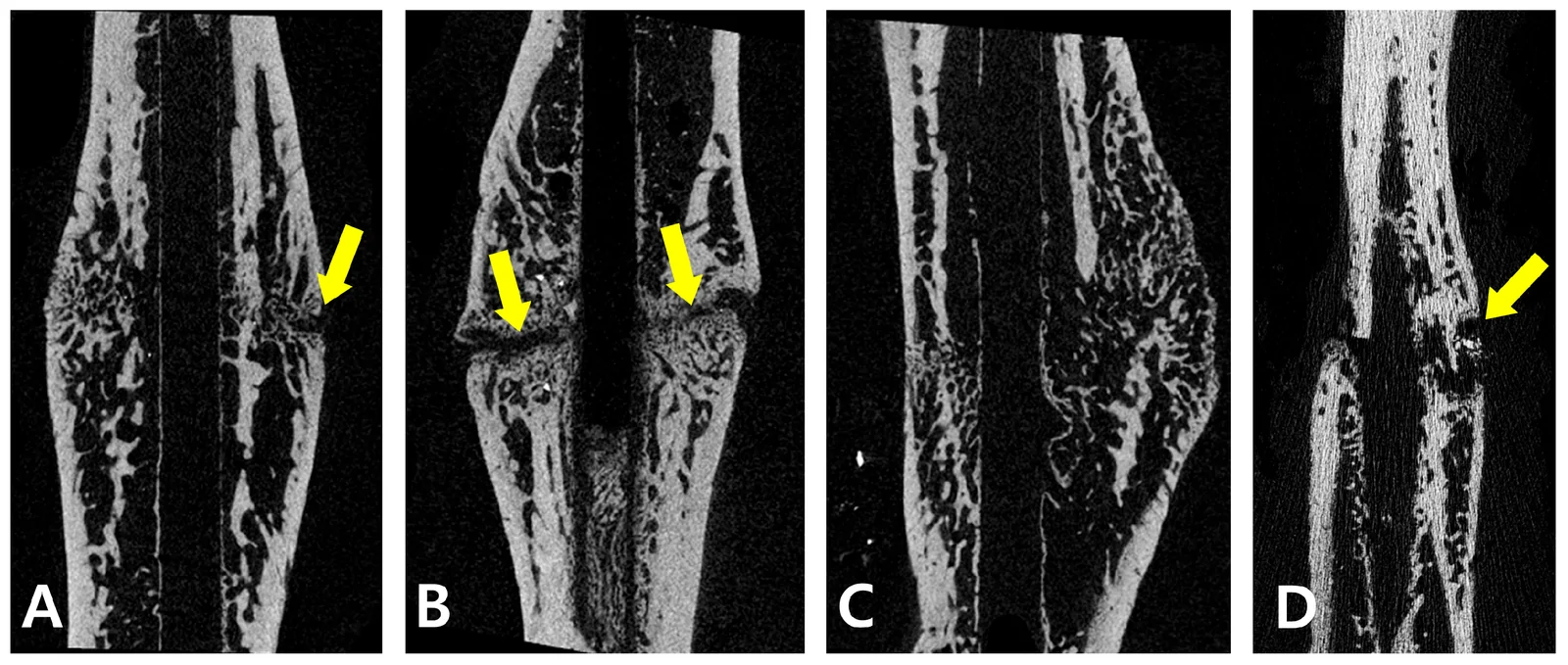
Micro-computed tomography cross-sectional image of rat femur six weeks after fracture. Fracture lines (yellow arrows) were observed (**A**) Group A (rats were injected with mesenchymal stem cells immediately post-fracture). Fracture lines (yellow arrows) suggest that fracture union is progressing but has not yet been completed. (**B**) Group B (rats were injected with mesenchymal stem cells 24 h post-fracture). Fracture union is progressing but a fracture gap is observed along the entire line of the fracture plane. (**C**) Group C (rats were injected with mesenchymal stem cells 7 days post-fracture). A greater volume of bone and fainter fracture gap were observed compared to figures (**A**,**B**,**D**). (**D**) Group D (control group) The fracture gap is narrowed but clearly observed with comparatively less new bone formation.

Micro-CT analysis showed mean PBV values of 11.9 ± 4.1%, 10.1 ± 4.8%, 20.5 ± 6.8%, and 10.7 ± 1.7% in groups A, B, C, and D, respectively. PBV was significantly higher in group C compared with that in groups A, B and D (*p* = 0.017, *p* = 0.003, *p* = 0.005, respectively). No significant difference was observed in PBV between groups A, B and D (*p* = 1.000) (Table 2, Figure 3). The effect size for group differences was medium (η^2^ = 0.29; 95% CI, 0.12–0.63). No animals were excluded or exhibited unexpected reactions. However, one sample in group C was lost during processing because of a handling error. A list of detailed PBV is shown in Appendix A Table A1.

### 3.2. Histological Fracture Healing Grades

**Figure 4:**
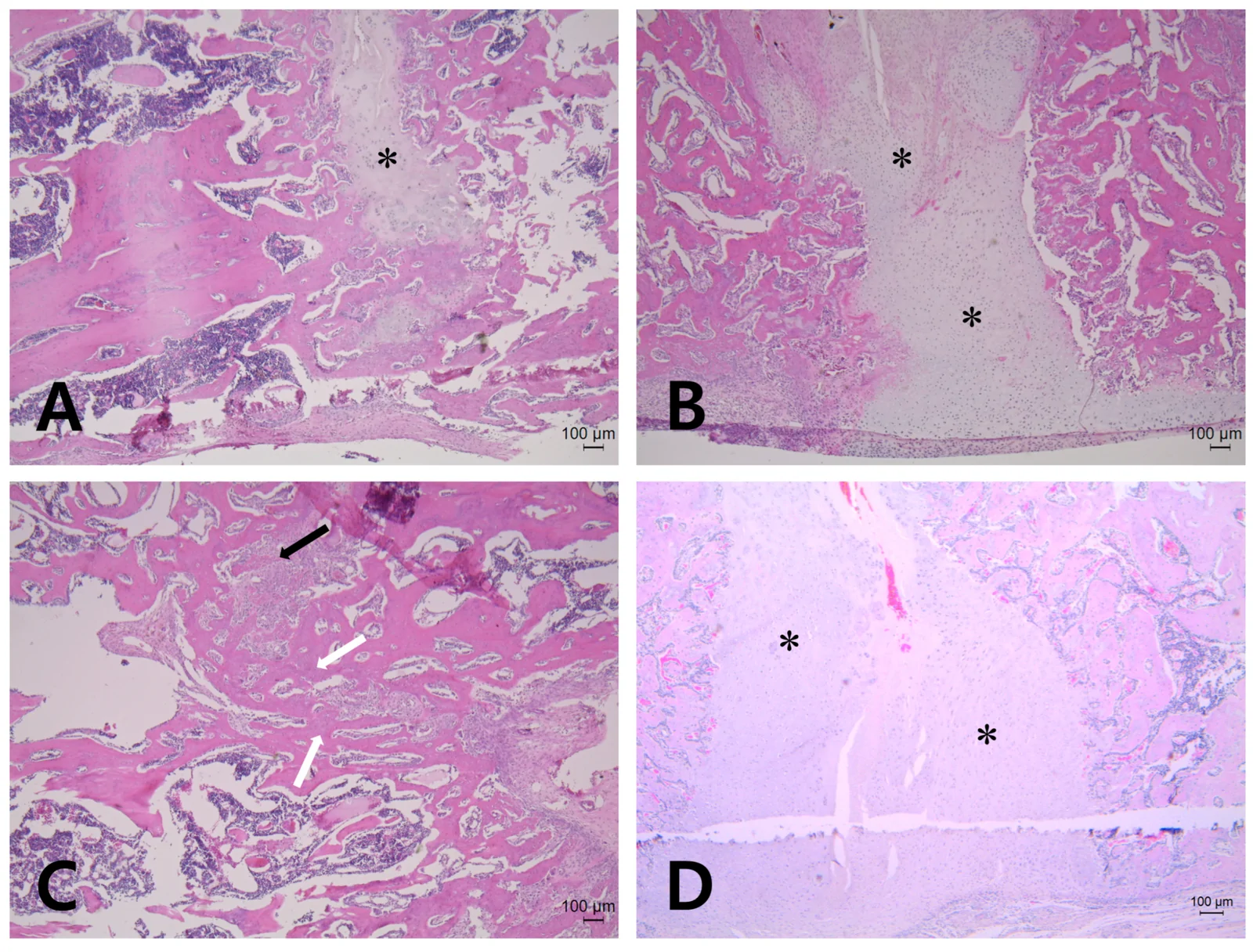
Hematoxylin and eosin staining of the tissue of fracture site (40× magnification) of rat femur six weeks after fracture. The black asterisks indicate fibro-cartilage tissue; the black arrow indicates immature bone; the white arrows indicate mature bone. (**A**) Group A (rats were injected with mesenchymal stem cells immediately post-fracture). Fibro-cartilage tissue is observed at the fracture site. (**B**) Group B (rats were injected with mesenchymal stem cells 24 h post-fracture) Fracture gap filled with fibro-cartilage tissue is shown. (**C**) Group C (rats were injected with mesenchymal stem cells 7 days post-fracture). Fracture gap is bridged by immature bone and mature bone. (**D**) Group D (control)**.** Wide fracture gap is filled with fibro-cartilage tissue.

**Figure 5:**
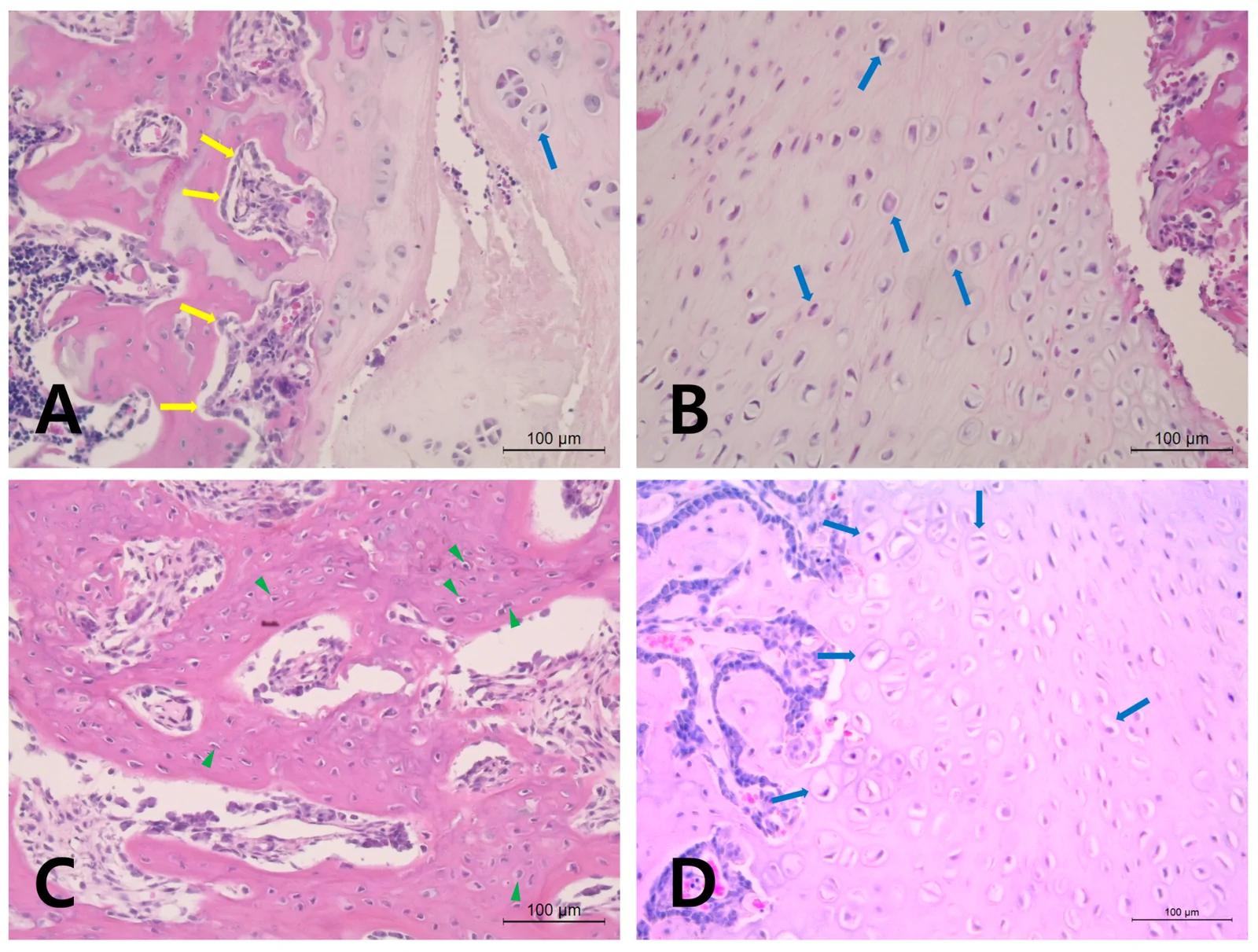
Hematoxylin and eosin staining of fracture site (200× magnification) of rat femur six weeks after fracture. The yellow arrows indicate osteoblasts; the blue arrows indicate chondrocytes with cartilage tissue; the green arrow heads indicate osteocytes in lacunae. (**A**) Group A (rats were injected with mesenchymal stem cells immediately post-fracture). Osteoblasts are arranged in a line at the junction of immature bone and cartilage. (**B**) Group B (rats were injected with mesenchymal stem cells 24 h post-fracture). The chondrocytes were observed in fibro-cartilage tissue. (**C**) Group C (rats were injected with mesenchymal stem cells 7 days post-fracture). Osteocytes housed within the lacunar structure of mature bone were observed. (**D**) Group D (control). The chondrocytes were observed in fibro-cartilage tissue.

Histological analysis showed fracture healing grades of 4.75 ± 3.1, 4.75 ± 1.26, 8.4 ± 2.19, and 4.1 ± 0.69 for groups A, B, C, and D, respectively. The Kruskal–Wallis test revealed a global statistical difference among the groups (*p* = 0.019). A significantly more mature bone was observed in group C compared with that in groups D (*p* = 0.018). Mann–Whitney U test revealed statistical difference in group C compared with group A and B (*p* = 0.045, *p* = 0.022, respectively). However, after Bonferroni adjustment, no significant difference was noted in group C compared with group A and B (*p* = 0.097, *p* = 0.349, respectively) (Table 3). The effect size for group differences was large (η^2^ = 0.43; 95% CI, 0.33–0.82). At 40× magnification, groups A, B, and D exhibited more fibro-cartilage tissue, whereas group C showed greater bony bridging of both immature and mature bones (Figure 4). At 200× magnification, fewer chondrocytes and more osteocytes were observed in group C [25,26] (Figure 5).

### 3.3. Western Blot

The amount of VEGF, TGF-β1, and BMP-2 was normalized by dividing each value by the β-actin level. As the protein level approached that of β-actin, the result approached 1.00.

**VEGF:** The mean VEGF protein level normalized by β-actin reached the highest value in group C (2.78 ± 1.21). Groups B (1.21 ± 0.25) and A (1.29 ± 0.82) followed with similar values. VEGF expression was significantly higher in group C than in groups A and B (*p* = 0.018, *p* = 0.016, respectively). No significant difference was found in VEGF expression between groups A and B (*p* = 1.000). The effect size for group differences was large (η^2^ = 0.44; 95% CI, 0.30–0.73). The pairwise comparison matrix of VEGF protein is delineated in Appendix A Table A2.

**TGF-β1:** The mean TGF-β1 protein level normalized by β-actin was high in groups C (1.89 ± 0.57) and B (1.76 ± 0.57), which showed similar values. The TGF-β1 protein level was the lowest in group A (1.01 ± 0.70). No statistically significant difference was noted between groups A, B, and C. However, the average was higher in the order of group C, B, and A. The effect size for group differences was large (η^2^ = 0.20; 95% CI, 0.00–0.66). The pairwise comparison matrix of TGF-β1 protein is listed in Appendix A Table A2.

**Figure 6:**
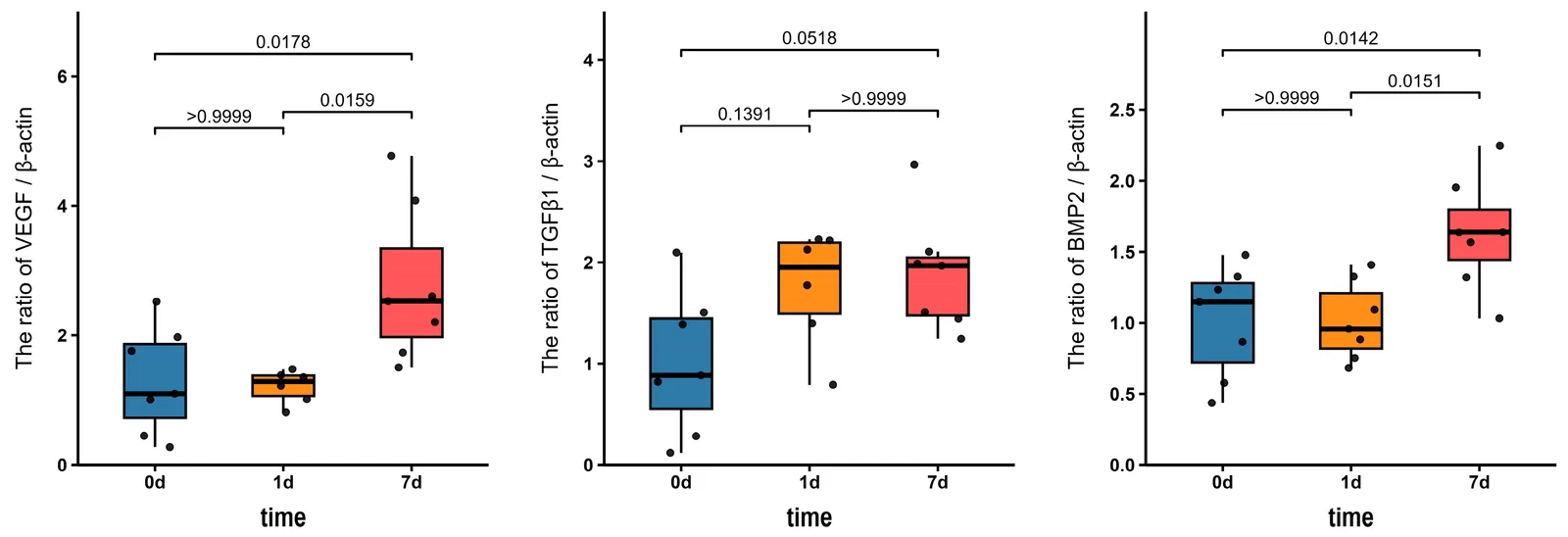
Relative expression levels of proteins related to angiogenesis [vascular endothelial growth factor (VEGF)], stem cell migration [transforming growth factor-beta 1 (TGF-β1)], and osteogenesis [bone morphogenetic protein-2 (BMP-2)].

**BMP-2:** The mean BMP-2 protein level normalized by β-actin reached the highest value in group C (1.63 ± 0.40). Groups B (1.01 ± 0.28) and A (1.01 ± 0.39) followed with similar values. BMP-2 expression was significantly higher in group C than in groups A and B (*p* = 0.014, *p* = 0.015, respectively). No significant difference was found in BMP-2 expression between groups A and B (*p* = 1.000) (Figure 6). The effect size for group differences was large (η^2^ = 0.34; 95% CI, 0.04–0.69). The pairwise comparison matrix of BMP-2 protein is delineated in Appendix A Table A2. A Original data of western blot is shown in Supplementary Table S1.

### 3.4. RT-qPCR

**VEGF:** The mean VEGF (VEGFA) mRNA expression level normalized by GAPDH was the highest in group C (4.55 ± 1.43), followed by group A (1.11 ± 0.49) and then group B (1.03 ± 0.43). Group C showed significantly higher VEGF expression than groups A and B (*p* < 0.001, *p* < 0.001, respectively). Groups A and B did not differ significantly (*p* = 1.000). The effect size for group differences was large (η^2^ = 0.62; 95% CI, 0.62–0.82). The pairwise comparison matrix of VEGF mRNA expression is listed in Appendix A Table A3.

**Figure 7:**
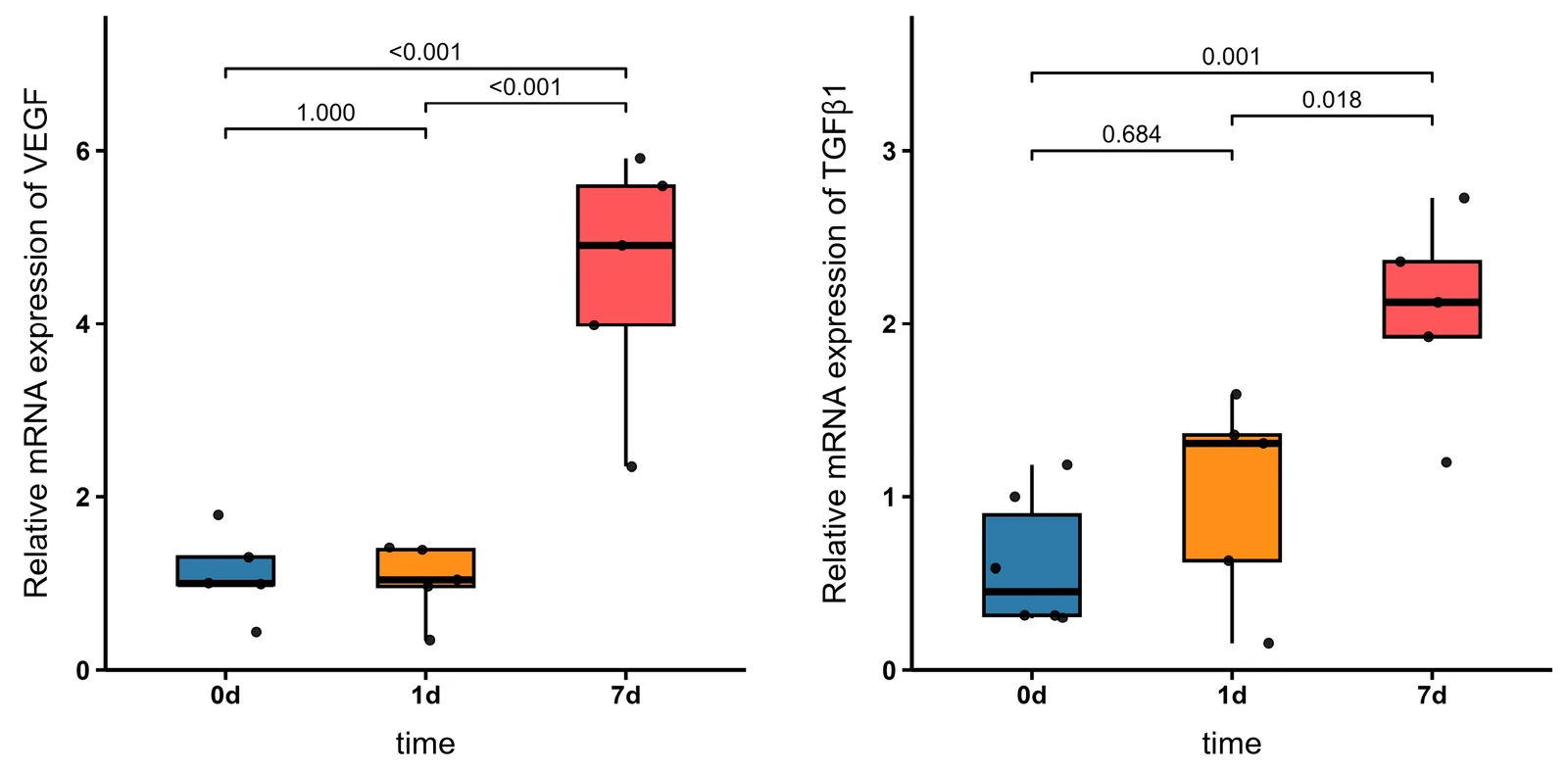
Relative mRNA expression levels of cytokines related to angiogenesis [vascular endothelial growth factor (VEGF)] and stem cell migration [transforming growth factor-beta 1 (TGF-β1)].

**TGF-β1:** The mean TGF-β1 mRNA expression level normalized by GAPDH was the highest value in group C (2.1 ± 0.57), followed by groups B (1.0 ± 0.60) and A (0.6 ± 0.39). Levels of TGF-β1 were significantly higher in group C than in groups A and B (*p* = 0.001, *p* = 0.018, respectively). No significant difference was found in TGF-β1 expression between groups A and B (*p* = 0.684) (Figure 7). The effect size for group differences was large (η^2^ = 0.53; 95% CI, 0.36–0.88). The pairwise comparison matrix of TGF-β1 mRNA expression is listed in Appendix A Table A3. A Original data of mRNA expression is shown in Supplementary Table S2.

## 4. Discussion

Fracture healing significantly enhanced in the 7-day group (group C) compared with the immediate group (group A), 24 h group (group B), and the control group (group D) when considering radiologic, histologic, and molecular analyses together.

Dreger et al. evaluated the homing effect of CD271-selected MSCs following systemic intravenous injection administered 24 h and 3 days after fracture in mice. They reported that 24 h after fracture showed the highest MSC migration. However, callus formation and callus size did not differ between the MSC-injected group and controls [11]. Wang et al. compared systemic intravenous stem cell injections at 24 h, 7 days, and 14 days after fracture and injected allogeneic MSCs into mice. They concluded that 7 days after fracture is the optimal timing for injection of bone-marrow-derived MSCs [12]. This study is meaningful as it is the first study to compare various MSCs injection timing including immediate injection.

### 4.1. Injection Timing of MSCs and Fracture Healing Process

The process of secondary fracture healing begins with an acute inflammatory response that peaks within the first 24 h and is complete after 7 days. This inflammatory phase features increased levels of cytokines such as tumor necrosis factor-α and interleu-kin-1. The reparative and remodeling phases follow [3,10,27]. This physiologic bone healing process suggests the hypothesis that can explain results of this study: MSCs injected at the beginning of the inflammatory phase suppress the physiologic inflammatory responses through their anti-inflammatory effects, which could hinder optimal healing. By contrast, MSCs injected 7 days after fracture act during the reparative and remodeling phases without inhibiting the inflammatory phase, which leads to enhanced bone healing [12].

The results of this study support the hypothesis that MSCs administered during the reparative phase, rather than during the acute inflammatory phase, provide the greatest benefit for bone regeneration. Further assessment methods, such as visualization of MSCs homing, should be investigated to develop this hypothesis into theory.

Wang et al. reported that stromal cell-derived factor-1 levels peak 1 week after fracture [12]. Stromal cell-derived factor-1 is a well-known chemokine that promote MSC migration through its interaction with chemokine receptor-4 on MSC surfaces.

The mechanisms through which MSCs enhance fracture healing remain incompletely understood. A hypothesis proposes that MSCs promote healing through direct differentiation into osteogenic cells such as osteoblasts. Although this mechanism has not been definitively proven, the reported homing and recruitment of MSCs to fracture sites provide indirect support. Another hypothesis suggests that cytokines secreted by MSCs, along with factors present in the extracellular matrix, mediate the pro-healing effects. Recent studies examining MSC-derived exosomes support this view [28,29]. The present study did not aim to clarify these mechanisms. But uncovering the mechanism of action is key in the journey of research to enhance fracture healing by stem cells.

### 4.2. Safety of Systemic MSC Application

Extensive research has established the ability of MSCs to enhance fracture healing. Recent investigations focus on determining the most effective method for MSC transplantation rather than reaffirming the already well-supported therapeutic effect.

Efforts to enhance fracture healing and bone formation through the application of MSCs have led to the development of several transplantation methods in animal studies, including the use of scaffolds [30], hydrogel mixtures, direct injection, and intravenous injection. Direct injection or placement of scaffolds combined with MSCs is the most commonly used transplantation method [4,31]. On the other hand, systemic injection such as intravenous or intracardiac injection is also a frequently reported method [32,33]. Intravenous injection has the advantage of not requiring opening the fracture site and enables flexible timing of injection. Concerns persist regarding potential systemic side effects following intravenous transplantation, but Ra et al. reported no systemic adverse effects and demonstrated the safety of systemic MSC injection in animals and humans [34]. Hou et al. infused osteocalcin-promoted MSCs intravenously. The expression of the osteocalcin transgene was restricted to osteoblasts and osteocytes [35].

Huang et al. compared the efficacy of intracardiac systemic injection and local injection at the fracture site using allogeneic bone-marrow-derived MSCs and reported the findings in 2015. In their study, 2 × 10^6^ cells were injected on day 4 post-fracture. Radio-graphic parameters and mechanical properties were significantly improved in both injection methods. However, no significant differences were observed between the systemic and local injection groups [20].

### 4.3. Optimal MSC Dosage for Fracture Healing

More than 5 million MSCs per rat were intravenously injected through one of the two tail veins. The optimal concentration of MSCs for intravenous injection in fracture models has not yet been established. A review of two studies using intravenous MSC injection in fracture models shows the variability in cell numbers. Dreger et al. collected human bone marrow during reaming of tibia intramedullary nailing. They isolated and cultured xenogeneic human bone-marrow-derived MSCs in their institute. The MSCs were trypsinized and counted for 2 × 10^6^ cells immediately before intravenous injection. The 2 × 10^6^ MSCs diluted in 200 µL of phosphate-buffered saline were injected via tail vein into mice [11]. Wang et al. used 1 × 10^6^ commercially supplied allogeneic rat MSCs suspended in PBS for tail vein injection into mice [12]. The number of stem cells administered via intravenous injection in these two studies appeared to be somewhat arbitrary.

Janko et al. inserted β-tricalcium phosphate scaffolds filled with xenogeneic human bone marrow mononuclear cells (BMCs) into large bone defects in rats and analyzed the effective BMC dose for bone healing. They identified a therapeutic window ranging from 1 × 10^6^ to 5 × 10^6^ BMCs, which they reported in 2020 [36]. Kim et al. investigated the optimal concentration of xenogeneic human MSCs for fracture healing by applying MSCs directly to the fracture site in a rat femoral shaft fracture model. In 2022, they reported that at least 5.0 × 10^6^ MSCs delivered to the fracture site is the optimal concentration to enhance therapeutic effects [4]. Although the MSC dose required for direct application cannot be directly compared with doses for intravenous injections, these studies guided the decision to administer 5.0 × 10^6^ MSCs intravenously in this study.

### 4.4. Why Choose Allogeneic MSCs Instead of Xenogeneic MSCs in This Study?

This study used fresh allogeneic bone-marrow-derived MSCs rather than human adipose-derived MSCs. Transplanted MSCs can be allogeneic or xenogeneic and isolated from various sources, including bone marrow, adipose tissue, dental pulp, skeletal muscle, liver, pancreas, nerves, and periosteum. Among these sources, human MSCs are commercially prepared and convenient to use. Their isolation and culture are optimized because of commercial demand. By contrast, isolation, purification, and cultivation of murine MSCs from bone marrow are relatively difficult due to their heterogeneity, low percentage in the bone marrow, and low yield, which makes expansion and harvesting difficult [15]. As a result, murine MSCs are seldom available commercially and are less accessible for experiment use. MSCs are immunosuppressive, and they do not elicit immediate immune responses [37,38]. This is the rationale for several fracture healing studies therefore applied human MSCs to rats instead of allogeneic rat MSCs. However, transplantation of human MSCs into rats carries an inherent risk of xenogeneic rejection. Therefore, in this study, we used fresh allogeneic bone-marrow-derived MSCs.

Most commercially available human MSCs are isolated from adipose tissue. In contrast, most murine MSCs used in research are derived from bone marrow. Obtaining MSCs from murine adipose tissue is feasible and detailed protocols of isolation and culture have been already introduced. However, the majority of studies continue to rely on bone-marrow-derived murine MSCs because they are more familiar to researchers and more frequently introduced.

Kim et al. applied xenogeneic human adipose-derived MSCs to rats [4]. Dreger at el. evaluated the migration of intravenously injected xenogeneic human bone-marrow-derived MSCs in mice in 2014 [11]. Janko at el. applied human bone marrow mono-nuclear cells (BMCs) obtained from German Red Cross into femoral defects in rats. BMCs include diverse cell types such as endothelial progenitor cells, hematopoietic stem cells, lymphocytes, and MSCs [36].

Wilson et al. evaluated bone defect regeneration in the ramus of swine with or with-out allogeneic swine adipose-derived MSC injections and reported the findings in 2012. They observed accelerated bone healing in a group that injected with MSCs [8]. Obermeyer et al. reported in 2012 that intravenous administration of allogeneic mouse MSCs increased callus volume and biomechanical strength in mouse model of alcohol-induced impaired fracture healing, which resulted in accelerated fracture healing [7].

### 4.5. Clinical Meaning and Limitations

Delayed fracture healing increases complications and social costs. Nonunion of a long bone shaft fracture represents a debilitating chronic medical condition that substantially harms health and quality of life [39]. Intramedullary nailing is the treatment of choice for femoral shaft fractures. However, even when performed by experienced sur-geons, nonunion rates in femoral shaft fractures range from 4% to 10% [40,41]. Humeral shaft fractures treated with intramedullary nailing show high complication and nonunion rates up to 33% [42,43]. The overall nonunion rate in human fractures is 4.9% [44]. Achieving fracture union in shaft fractures of long bones is important for improving patient prognosis.

This study has several limitations. First, molecular biologic evaluation methods could not include control group (group D), because number of sample throughput that could be processed simultaneously is limited by equipment capacity. Therefore, the molecular data only permit comparisons among MSC-treated groups and cannot demonstrate increased expression compared to physiologic fracture healing. But radiologic and histological analysis was conducted for control group and the results showed that the fracture healing was enhanced in 7 days after fracture group (group C).

A second limitation is the arbitrariness of injection timing selection. Limited number of studies investigated the timing of intravenous MSC administration for fracture healing. Dreger et al. evaluated MSC homing following systemic injection at 24 h and 3 days post-fracture in mice and identified 24 h as the optimal timing [11]. Wang et al. compared tail vein injection at 1, 7, and, 14 days post-fracture in a murine model and concluded that 7 days was optimal [12]. Based on the optimal injection timings determined in these studies, the present study added an immediate post-fracture injection group, resulting in four comparative groups: immediate injection, 1 day after fracture, 7 days after fracture, and control group. However, the selection of injection timings remains somewhat arbitrary.

A third limitation relates to the evaluation methods used, which required euthanasia and excision of the femur. Therefore, the progression of fracture healing over time could not be observed. Interim micro-CT in rats can be evaluated under anesthesia, which pro-vides insights into the progression of fracture healing, but it was not included in this study.

A fourth limitation is that evaluation methods could be applied more. The MSCs migration was not graphically visualized using fluorescence imaging. If these analyses were conducted, the mechanism of fracture healing enhancement would be less speculative. If the inflammatory cytokines such as IL-1 or TNF-α were analyzed, it could be helpful to support hypothesis can explain result of this experiment. There is a lack of commonly used osteogenic markers analysis, such as RUNX2, OCN, ALP, COL1A1, and OPN. Additional specific stains that could enhance the evaluation of histologic features exist [45].

Finally, clinical translation remains challenging because fracture healing progresses at different rates in humans and rats. Rat femoral fractures typically heal within 6 weeks, whereas humans require a significantly longer period, ranging from 6 to 9 months [46]. Despite these limitations, this study provides meaningful insights and may support the development of systemic intravenous stem cell treatments that can be widely used even for conservative management of closed fractures.

## 5. Conclusions

Systemic intravenous MSC administration enables flexible selection of injection timing regardless of fracture occurrence. Among the timings examined (immediate injection, 24 h after fracture, and 7 days after fracture), 7 days after fracture was the optimal timing to enhance fracture healing. These findings indicate that MSC therapy administered on day 7 aligns with the reparative phase and maximizes fracture healing in rats. Further research like functional mobility tests should be encouraged to reinforce optimal timing of MSCs administration for enhancing fracture healing.

## Figures and Tables

**Table 1:** Primers used for quantitative real-time polymerase chain reaction.

Gene	Forward Primer (5′–3′)	Reverse Primer (5′–3′)
*VEGF*	GTACCTCCACCATGCCAAGT	AATAGCTGCGCTGGTAGACG
*TGF-β1*	ACTACGCCAAAGAAGTCACC	CCGAATGTCTGACGTATTGAAGA
*GAPDH*	GGCAAGTTCAACGGCACAG	CGCCAGTAGACTCCACGACA

*VEGF*, vascular endothelial growth factor; *TGF-β1*: transforming growth factor-beta 1; *GAPDH*, glyceraldehyde 3-phosphate dehydrogenase.

**Table 2:** Radiologic evaluation of fracture healing using micro-computed tomography.

MSCs Injection Timing	Immediate Group A	Post-Fracture 1 Day Group B	Post-Fracture 7 Days Group C	Control Group D
Percentage bone volume, (%)	11.9 ± 4.1	10.1 ± 4.8	20.5 ± 6.8	10.7 ± 1.7
Group A	-	1.000	0.017	1.000
Group B	1.000	-	0.003	1.000
Group C	0.017	0.003	-	0.005
Group D	1.000	1.000	0.005	

Statistical analysis was performed using one-way ANOVA, followed by Bonferroni post hoc tests. η2 = 0.29; 95% CI 0.12–0.63.

**Table 3:** Histological grading of fracture healing process.

MSCs Injection Timing	Immediate Group A	Post-Fracture 1 Day Group B	Post-Fracture 7 Days Group C	Control Group D
Average grading	4.75 ± 3.1	4.75 ± 1.3	8.4 ± 2.2	4.1 ± 0.69
Group A	-	1.000	0.097	1.000
Group B	1.000	-	0.349	1.000
Group C	0.097	0.349	-	0.018
Group D	1.000	1.000	0.018	-

Statistical analysis was performed using Kruskal–Wallis method, followed by Bonferroni post hoc tests. η^2^ = 0.43; 95% CI 0.33–0.82.

## Data Availability

The raw data supporting the conclusions of this article will be made available by the authors on request.

## Supplementary Materials

The following supporting information can be downloaded at: https://www.mdpi.com/article/10.3390/jfb17080361/s1. Table S1: Original data of western blot; Table S2: Original data of mRNA expression.

## Abbreviations

The following abbreviations are used in this manuscript:

MSCs	Mesenchymal stem cells
CD	Cluster of differentiation
micro-CT	Micro-computed tomography
PBV	Percentage bone volume
VEGF	Vascular endothelial growth factor
TGF-ß1	transforming growth factor-beta 1
BMP-2	Bone morphogenetic protein-2
RT-qPCR	Reverse transcription quantitative polymerase chain reaction

## Appendix A

**Table A1:** Detailed percentage bone volume of each rat.

Rats ID	Immediate Group A	Post-Fracture 1 Day Group B	Post-Fracture 7 Days Group C	Control Group Group D
Rat A1, B1, C1, D1	14.9256	6.2420	12.9768	12.7410
Rat A2, B2, C2, D1	14.6380	13.3502	22.3452	10.3130
Rat A3, B3, C3, D1	12.4302	5.6225	22.4772	7.4388
Rat A4, B4, C4, D1	17.6183	11.8240	22.2487	11.3313
Rat A5, B5, C5, D1	7.0657	17.8227	12.3520	11.9561
Rat A6, B6, C6, D1	7.4698	11.0010	30.4972	10.1606
Rat A7, B7, C7, D1	9.2656	4.5344	Not Available	10.9368

Percentage bone volume (%).

**Table A2:** (**2-a**) Pairwise group comparison of VEGF by Western blot. (**2-b**) Pairwise group comparison of TGF-β1 by Western blot. (**2-c**) Pairwise group comparison of BMP-2 by Western blot.

2-a			
VEGF	Immediate Group A	Post-fracture 1 day Group B	Post-fracture 7 days Group C
Average	1.29 ± 0.82	1.21 ± 0.25	2.78 ± 1.21
Normality *p*-value	0.772	0.532	0.290
Group A	-	1.000	0.018
Group B	1.000	-	0.016
Group C	0.018	0.016	-
2-b			
TGF-β1	Immediate Group A	Post-fracture 1 day Group B	Post-fracture 7 days Group C
Average	1.01 ± 0.70	1.76 ± 0.57	1.89 ± 0.57
Normality *p*-value	0.836	0.188	0.381
Group A	-	0.139	0.052
Group B	0.139	-	0.100
Group C	0.052	0.100	-
2-c			
BMP-2	Immediate Group A	Post-fracture 1 day Group B	Post-fracture 7 days Group C
Average	1.01 ± 0.39	1.01 ± 0.28	1.63 ± 0.40
Normality *p*-value	0.558	0.842	0.935
Group A	-	1.000	0.014
Group B	1.000	-	0.015
Group C	0.014	0.015	-

VEGF, vascular endothelial growth factor. η^2^ = 0.44; 95% CI, 0.30–0.73; Homoscedasticity *p*-value 0.140. TGF-β1, transforming growth factor-beta 1. η^2^ = 0.20; 95% CI, 0.00–0.66; Homoscedasticity *p*-value 0.812. BMP-2, bone morphogenetic protein 2. η^2^ = 0.34; 95% CI, 0.04–0.69; Homoscedasticity *p*-value 0.753.

**Table A3:** (**3-a**) Pairwise group comparison of VEGF mRNA expression. (**3-b**) Pairwise group comparison of TGF-β1 mRNA expression.

3-a			
VEGF	Immediate Group A	Post-fracture 1 day Group B	Post-fracture 7 days Group C
Average	1.11 ± 0.49	1.03 ± 0.43	4.55 ± 1.43
Normality *p*-value	0.881	0.304	0.710
Group A	-	1.000	<0.001
Group B	1.000	-	<0.001
Group C	<0.001	<0.001	-
3-b			
TGF-β1	Immediate Group A	Post-fracture 1 day Group B	Post-fracture 7 days Group C
Average	0.6 ± 0.37	1.0 ± 0.60	2.1 ± 0.57
Normality *p*-value	0.072	0.398	0.892
Group A	-	0.684	0.001
Group B	0.684	-	0.018
Group C	0.001	0.018	-

VEGF, vascular endothelial growth factor. η^2^ = 0.62; 95% CI, 0.62–0.82; Homoscedasticity *p*-value 0.135. TGF-β1, transforming growth factor-beta 1. η^2^ = 0.53; 95% CI, 0.36–0.88; Homoscedasticity *p*-value 0.809.
