# Supplementary material for: Timing for Intravenous Injection of Mesenchymal Stem Cells to Enhance Fracture Healing
Source: J Funct Biomater. 2026 Jul 27;17(8):361. doi: 10.3390/jfb17080361 (PMC13514731; doi:10.3390/jfb17080361)
Supplement: Supplementary file 1 [file jfb-17-00361-s001.zip › jfb-4344966-supplementary.pdf]

Supplementary Table S1-1. Original data of western blot of VEGF

Normalized value by β-actin

Rats ID	Immediate<br>Group A	Post-fracture 1 day<br>Group B	Post-fracture 7 days<br>Group C
Rat A1, B1, C1	0.447	1.354	4.079
Rat A2, B2, C2	0.273	0.811	4.776
Rat A3, B3, C3	1.007	1.387	2.604
Rat A4, B4, C4	1.098	1.012	1.503
Rat A5, B5, C5	1.969	1.219	2.529
Rat A6, B6, C6	1.757	1.476	1.735
Rat A7, B7, C7	2.521	Sample loss	2.208

Supplementary Table S1-2. Original data of western blot of TGF β1

Normalized value by β-actin

Rats ID	Immediate<br>Group A	Post-fracture 1 day<br>Group B	Post-fracture 7 days<br>Group C
Rat A1, B1, C1	0.118	2.217	1.967
Rat A2, B2, C2	0.283	1.400	1.990
Rat A3, B3, C3	2.098	2.128	1.445
Rat A4, B4, C4	0.883	0.790	1.507
Rat A5, B5, C5	1.504	1.774	2.965
Rat A6, B6, C6	0.820	2.228	2.107
Rat A7, B7, C7	1.389	Sample loss	1.245

Supplementary Table S1-3. Original data of western blot of BMP-2

Normalized value by β-actin

Rats ID	Immediate<br>Group A	Post-fracture 1 day<br>Group B	Post-fracture 7 days<br>Group C
Rat A1, B1, C1	0.436	1.408	1.637
Rat A2, B2, C2	1.325	1.326	2.247
Rat A3, B3, C3	1.234	0.957	1.320
Rat A4, B4, C4	0.865	1.092	1.954
Rat A5, B5, C5	1.478	0.682	1.567
Rat A6, B6, C6	1.147	0.882	1.639
Rat A7, B7, C7	0.577	0.753	1.032

Supplementary Table S2-1. Original data of mRNA expression of VEGF

Normalized value by GAPDH

Rats ID	Immediate<br>Group A	Post-fracture 1 day<br>Group B	Post-fracture 7 days<br>Group C
Rat A1, B1, C1	1	Error	5.588
Rat A2, B2, C2	Error	1.412	Error
Rat A3, B3, C3	1.302	0.966	Error
Rat A4, B4, C4	1.797	1.042	4.909
Rat A5, B5, C5	0.437	0.339	5.912
Rat A6, B6, C6	0.988	1.388	2.354
Rat A7, B7, C7	Sample loss	Sample loss	3.987

Supplementary Table S2-2. Original data of mRNA expression of TGF-β1

Normalized value by GAPDH

Rats ID	Immediate<br>Group A	Post-fracture 1 day<br>Group B	Post-fracture 7 days<br>Group C
Rat A1, B1, C1	0.95	Error	1.924
Rat A2, B2, C2	Error	1.357	Error
Rat A3, B3, C3	0.317	1.31	1.201
Rat A4, B4, C4	0.358	1.594	2.125
Rat A5, B5, C5	0.301	0.63	2.357
Rat A6, B6, C6	1.186	0.154	Error
Rat A7, B7, C7	0.587	Sample loss	2.726
